# Changes of Inertial Focusing Position in a Triangular Channel Depending on Droplet Deformability and Size

**DOI:** 10.3390/mi11090839

**Published:** 2020-09-07

**Authors:** Yo-han Choi, Jeong-ah Kim, Wonhee Lee

**Affiliations:** 1 Graduate School of Nanoscience and Technology, Korea Advanced Institute of Science and Technology (KAIST), Daejeon 34141, Korea; cyhan308@kaist.ac.kr; 2 Department of Physics, KAIST, Daejeon 34141, Korea; jeongah_nano@kaist.ac.kr; 3 Department of Bio and Brain Engineering, KAIST, Daejeon 34141, Korea

**Keywords:** inertial focusing, deformability, triangular channel

## Abstract

Studies on cell separation with inertial microfluidics are often carried out with solid particles initially. When this condition is applied for actual cell separations, the efficiency typically becomes lower because of the polydispersity and deformability of cells. Therefore, the understanding of deformability-induced lift force is essential to achieve highly efficient cell separation. We investigate the inertial focusing positions of viscous droplets in a triangular channel while varying Re, deformability, and droplet size. With increasing Re and decreasing droplet size, the top focusing position splits and shifts along the sidewalls. The threshold size of the focusing position splitting increases for droplets with larger deformability. The overall path of the focusing position shifts with increasing Re also has a strong dependency on deformability. Consequently, droplets of the same size can have different focusing positions depending on their deformability. The feasibility of deformability-based cell separation is shown by different focusing positions of MCF10a and MCF7 cells.

## 1. Introduction

Diverse microfluidic particle manipulation methods have been developed to separate, enrich, or capture particles and cells [1,2,3,4]. Inertial microfluidics is a highly efficient technique for controlling micro-size particles within fluid flows in relatively inexpensive and straightforward methods without external forces [5,6,7,8]. Within finite-Reynolds-number (finite-Re) flows, suspended particles migrate across the mainstream toward equilibrium positions where the wall effect lift force and shear gradient lift force are balanced. The equilibrium positions, or focusing positions, change with many parameters. Flow parameters (flow speed and viscoelasticity [9,10,11]), channel parameters (cross-sectional shape, aspect ratio, and channel curvature [12,13,14,15,16]), and particle parameters (size, shape, and deformability [17,18,19]) can be tuned to modulate the number and location of inertial focusing positions.

In particular, many interesting results have been reported with triangular cross-section channels [12,13,14]. The number and location of focusing positions are found to vary depending on the vertex angle, particle size, and Re. Notably, interesting splitting and shifting of the top focusing position are found and studied in depth. It is believed that the changes of the top focusing position are resulted by the two lift forces (i.e., shear-gradient lift force and wall effect lift force) being not in a parallel direction, unlike rectangular channels. As Re increases, the top focusing position that initially locates near the vertex splits and shifts along the sidewalls. The onset of the splitting and the distance of the focusing position shift are strongly dependent on the particle size, which enables highly efficient particle and cell separations. 

Various inertial microfluidic devices have been developed for the separation and enrichment of cells. However, in most cases, the focusing and separation efficiency for cells is significantly lower than those of solid particles [4,13,20]. The decrease in efficiency stems from two primary sources: polydispersity and deformability. First, cells are naturally polydisperse. While solid particles used for experiments are prepared with small coefficients of variation (C.V. < 5%), cells typically have a wide size distribution, which makes it difficult to separate them entirely based on size. Second, cells can deform under stress by surrounding fluid flows, which induces deformability-induced lift force. Deformability is one of the crucial parameters that change the inertial focusing positions of cells [18]. It has been reported that particles of larger deformability generally have focusing positions closer to the channel center. Therefore, the optimized condition for the separation of solid particles cannot be directly used for cell separations, and often a mixture of cells with varying deformability complicates the separation.

We investigated the inertial focusing of droplets in a triangular channel to understand how the deformability of particle changes the focusing positions. The focusing positions of viscous oil droplets are studied in a microchannel with a right isosceles triangular cross-section while varying Re and droplet size. By combining the observation from the top view and the side view, the splitting and shifting path of the top focusing position as a function of Re are plotted in the channel cross-section, which shows dependency on the deformability and the size of droplets. Especially, the focusing positions of droplets with different deformability become clearly distinguishable at large Re. The deformability of cells can vary depending on cell types and their phenotype, and it is used as a biomarker [21,22,23]. We also show that the cells with different deformability can have different focusing positions, which suggests that the triangular channels can be used for a deformability-based inertial separation of cells.

## 2. Background, Materials, and Methods

### 2.1. Inertial Focusing

Inertial lift forces in a microchannel allow particles and cells to focus at a specific cross-sectional position without external forces [8,11,24]. The focusing positions are mainly determined by the balance of wall effect lift force and shear gradient lift force. Near the channel wall, the wall effect lift force acts dominantly. Vortices formed on a particle surface develop asymmetrically due to the wall effect. As the distance between the wall and the particle becomes small, the fluid between them will accelerate, and lift force arises in the direction away from the wall [25]. The shear gradient lift force arises due to the curvature in the velocity profile. The shear gradient lift force acts strongly over the entire area in the channel. In a typical microfluidic channel with a parabolic velocity profile, the shear gradient lift force pushes particles down the gradient of shear rate—that is, away from the channel center [26,27]. The inertial focusing position by the balance of the two lift forces forms typically at approximately 0.6 times the radius from the channel center [28]. The direction of the shear gradient lift force is determined by the product of shear rate and its gradient. With a complex velocity profile, the direction of the shear gradient lift force can be toward the channel center, and the focusing positions can form at inflection points of the velocity profile. This inflection point focusing has been demonstrated in co-flows of fluids with different viscosity [29,30].

Studies using rectangular channels revealed that the inertial focusing position could change depending on various factors, including particle size, shape, channel aspect ratio, and Re [5,11,31]. In square channels, the focusing positions are found near the center of each face. In rectangular channels, the focusing positions are generally found near the long faces. However, it can change depending on particle size and Re; the unstable focusing positions can be stabilized when the particle size is small or the Re is high, and the focusing position can form on all four faces [32,33]. The focusing position can move closer to channel walls with decreasing particle size and the larger Re. Various studies have been conducted for the separation of particles based on size. Many parameters can affect the focusing positions, and they have been investigated to maximize the difference of focusing positions depending on the particle size. The difference of the focusing position for different size particles is relatively small in straight rectangular channels, and many different channel geometries and combinations of different forces such as viscoelastic force have been extensively investigated.

### 2.2. Inertial Focusing in Triangular Channels

**Figure 1:**
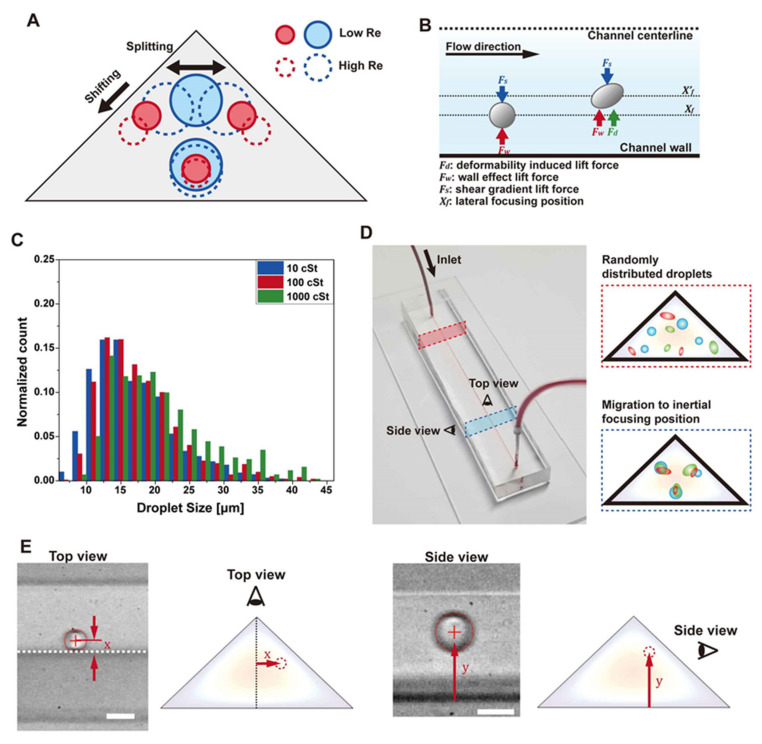
Overview of background and experiments. (**A**) Inertial focusing positions in an equilateral right triangle channel. The increase in Re and decrease in particle size lead to the top focusing position splitting and shifting. (**B**) Inertial focusing of deformable particles. Deformable particles are subjected to additional deformability-induced lift forces toward the center of the channel. (**C**) Size distribution of oil droplets. (**D**) Summary of the experimental method. Deformable oil droplets with various size are flowed in a triangular microchannel. Particles near the outlet are focused at equilibrium positions and observed from the top view and the side view. (**E**) Examples of measurements from the top view and side view. Scale bar: 20 μm.

The cross-sectional shape of a microchannel determines its velocity profile, which obviously can influence the inertial focusing positions. Triangular channels display unusual inertial focusing due to their unique velocity profile and the angles between channel walls [13,14]. The focusing positions change depending on particle size, vertex angles, and Re. The corner focusing potions are reported for large particles at relatively low Re. Otherwise, focusing positions generally appear close to all three channel faces. A recent publication reported that the details of the focusing position change depending on the vertex angles [13]. In the case of an equilateral triangle, the focusing positions appear near the center of each face, which does not shift with Re due to the symmetry of the channel cross-section. In the case of acute triangles, the focusing position near the bottom channel wall becomes unstable at low Re, and the two focusing positions near sidewalls (top focusing positions) shift upward with increasing Re. The bottom focusing position reappears at high Re. In the case of obtuse triangles, the top focusing positions shift downward along the sidewalls, while the bottom focusing position remains unchanged. When the particle size is large, the top focusing positions can merge into the top corner focusing positions. To summarize the changes in obtuse triangular channels, the top focusing position appears as one near the apex if the Re is low and particle size is large, and it splits and shifts downward along the sidewall with increasing Re or decreasing particle size, as shown in Figure 1A. The onset Re of the splitting is strongly dependent on the size of particle; therefore, the top focusing positions become significantly different for different size particles at given Re. This property allows a highly efficient size-based cell separation [13].

### 2.3. Deformability-Induced Lift Force

Deformable particles can experience lateral migration under Poiseuille flows, as shown in Figure 1B. Cross-stream migrations of bubbles [34], droplets [35], and vesicles [36] in microchannels have been studied, and it is known that the cross-stream migration of such deformable particles can also occur at very low Re [37]. Deformable particles can be in different types, including elastic solids, capsules, and droplets. In addition to the lift force generated by deformed particle shapes, nonlinearities caused by the matching of velocities and stresses at the interface can lead to deformability-induced lift force. In this case, We (*ρ*$U^{2}a/\sigma$, where ρ is the density of fluid, U is the average fluid velocity, a is the particle diameter, and σ is the surface tension), Ca ($\mu Ua/\sigma D_{h}$, where µ is the dynamic viscosity of fluid and $D_{h}$ is the hydraulic diameter), and the relative viscosity ratio between internal and external fluid become essential parameters for understanding the lift force [38]. In most cases, deformability-induced lift force pushes particles toward the center of the channel, and the focusing position shifts toward the channel center as the viscosity decreased. However, if the viscosity decreases below 5 cSt, the focusing position moves back toward the channel wall again [18,38]. Microparticles or cells can be separated depending on the deformability using this difference in focusing position using a straight channel [18] and spiral channel [39].

The deformability of cells has been studied by various methods. For example, cells can be flowed in microchannels to deform under shear flow [18,40,41,42], or cells can be pushed/pulled into a small gap (e.g., pipette tip or microchannel structure) [43,44,45]. In the case of inertially focused cells, cells are deformed by the strong shear stress of the parabolic velocity profile, which causes the cells to elongate, similar to a droplet. Such deformation leads to the deformability-induced lift force. To study the relationship between cell deformability and inertial focusing position, one needs to analyze the deformations under shear flows and resulting deformability-induced lift force. Hur et al. showed that the deformation of cells in an inertial microfluidic flow can be studied adequately by viscous droplets [18].

### 2.4. Oil Droplet Preparation

Deformable droplets are generated with silicon oil of viscosity of 10, 100, and 100 cSt (Sigma Aldrich, St. Louis, MI, USA) using a vortex mixer (VM-10, WiseMix, Daegu, Korea). Deionized water (9 mL) and silicone oil (1 mL) is mixed, and tween 20 (3% *w/v*, Sigma Aldrich, St. Louis, MI, USA) is added. The mixture is mixed vigorously with the vortex mixer for approximately 2 min. Then, the mixture is left for approximately 3 min so that relatively large drops can float on top of the water. After removing the top portion of the mixture, droplets less than the diameter of approximately 30 μm can be collected, as shown in Figure 1C. We use 3% *w/v* concentration of tween 20, which is higher than the critical micelle concentration. It can be assumed that the surface of the droplet is sufficiently saturated with the surfactant molecule [18].

### 2.5. Cell Preparation

MCF7 cells (a breast carcinoma cell line) were cultured with Dulbecco’s Modified Eagle’s Medium (DMEM, WELGENE, Daegu, Korea) including 10% *v/v* Fetal Bovine Serum (FBS, WELGENE, Daegu, Korea) and 1% *v/v* penicillin-streptomycin solutions (WELGENE, Daegu, Korea) and incubated at 37 °C and 5% CO_2_ concentration using a CO_2_ incubator (Sanyo, Osaka, Japan). MCF10a cells (an immortalized breast epithelial cell line) were cultured in growth media suggested by The American Type Culture Collection (ATCC, Bethesda, MD, USA).

### 2.6. Device Fabrication

The polydimethylsiloxane (PDMS) microfluidic channel is fabricated by soft lithography from a mold with a triangular cross-section. The triangular cross-section mold is fabricated with UV glue (NOA71, Norland, Fort Atkinson, WI, USA) replicated from a brass mold that is made with a planing process [12,13,46]. The brass workpiece is scratched with a V-shaped diamond cutting tool with a 90-degree vertex angle to make triangular grooves. PDMS (Sylgard 184, Dow Corning, Midland, MI, USA) is mixed in a 1:10 ratio of base and cross-linker and poured into the brass mold to make a PDMS mold. This PDMS replica can be directly used as a PDMS channel mold. However, the PDMS mold is easily damaged during repeated uses. The first PDMS channel from the PMDS mold is used to fabricate a UV glue mold. The PDMS channel is placed on a silicon wafer, and the channel is filled with UV glue. The UV glue is cured under the exposure of a UV lamp (365 nm, UVITEC CAMBRIDGE, Cambridge, UK). Then, PDMS channels are fabricated from this UV glue mold by conventional soft lithography and plasma bonding. A PDMS mixture is poured on the UV glue mold and cured for 2 h at 65 °C. The cured channel is cut out, and the inlet/outlet are punched with the biopsy punch (Darwin microfluidics, Paris, France) subsequently. The PDMS channel is bonded to slide glass using oxygen plasma (PDC-002, Harrick Plasma, Ithaca, NY, USA). The final channel cross-section has the shape of a right-angled isosceles triangle with the bottom of 100 μm and the height of 50 μm, and the channel length is approximately 4 cm. The devices for obtaining side-view images are made separately, as described in a previous study [12].

### 2.7. Image Analysis

Droplets are flowed through triangular channels, and high-speed capture images are obtained near the outlet from the top view and the side view using a microscope (Nikon Eclipse Ti-U, Nikon, Tokyo, Japan) equipped with a high-speed camera (Phantom v7.3, Wayne, NJ, USA) (Figure 1D,E). High-speed capture images are analyzed for particle position and size. The x and y positions are measured in the top view and side view image. The center positions and the size of each droplet are measured using customized MATLAB code in a way to detect boundaries by sensing the intensity gradient. The droplet size is defined as the diameter of the sphere without deformation. It is challenging to know the exact 3-dimensional shape of a deformed droplet, because the image is only taken in one direction. We define the diameter of the droplet as the average length in the x-direction and the y-direction. Taking the diameter of the sphere in this manner is expected to result in small errors. For example, when ignoring depth information and only comparing areas, the area of the ellipse with eccentricity of 0.8 has an error of 5% compared to the area of the circle whose diameter is the average length in the x and y direction from the ellipse center. Most droplets observed in the experiment have an eccentricity of 0.8 or less, which can contribute <5% error. This error by the uncertainty of the 3D shape corresponds to <1 μm error for a 20 μm droplet. Measurement of the droplet size from high-speed images has several intrinsic errors from image blur. Especially, we have a considerable variation of the droplet positions and size. We filter out the out-of-focus images of droplets so that only particles near the focal plane can be analyzed. The width of the boundary of the droplet image sets the upper limit of the error in size measurement. We have < ±1 μm of errors in locating the true boundary, which is comparable to the errors resulting from the uncertainty of the 3D shape.

## 3. Results

### 3.1. Deformation of Viscous Oil Droplet

**Figure 2:**
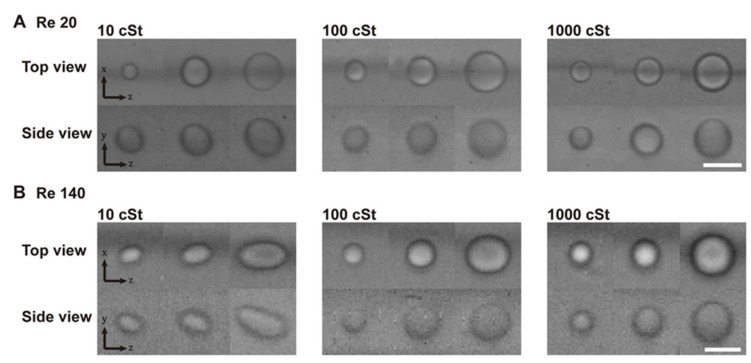
Deformation of oil droplets. High-speed images of oil droplets of various viscosities and sizes. (**A**) Low Re condition (Re = 20). Droplet deformation is generally insignificant. Scale bar: 20 μm. (**B**) High Re condition (Re = 140). Elongation of the droplets is clear for 10 cSt droplets, and slight deformation is observed for 100 cSt droplets. Scale bar: 20 μm.

First, we observe the deformation of droplets under the shear flow formed within the triangular channel shown in Figure 2. The deformability-induced lift force can be estimated to some extent by the change in the droplet shape. Droplets with a smaller viscosity are expected to show more significant changes with increasing Re. Re is calculated as$\;\rho UD_{h}/\mu$. Re is controlled by adjusting the flow rate, which changes the average fluid velocity U. The droplets with a viscosity of 10, 100, and 1000 cSt are observed from the top view and the side view. The range of droplet viscosity is chosen to investigate the different degrees of deformation following the previous study [18]. In this reference, the inertial focusing positions of cells were found to change depending on both the size and the deformability. The focusing positions of cells were located mostly between the focusing positions of the droplets with a viscosity ranging from approximately 5 cSt to 1000 cSt. Figure 2 shows representative images of droplets of three different sizes near their focusing positions at Re = 20 (Figure 2A) and Re = 140 (Figure 2B). At Re = 140, the images become noisy because of the short exposure time that is limited by high down-stream speed. Note that the images from the side view are noisier because the illumination light intensity is reduced due to the structure of the side view device. At the low flow rate (Re = 20), droplets with all viscosity show very small or no noticeable deformation. There is a small elongation observed only for 10 cSt droplets in the side views. As the flow rate increases (Re = 140), droplets become more responsive to the shear gradient, and the shape changes become clearly observed. Small deformation can be observed for 100 cSt. Yet, little deformation is observed for 1000 cSt droplets. The deformed shape under a parabolic flow profile is as shown in Figure 1B, which is similar to the deformation seen from the side view. Note that the droplets are located above the channel centerline here. In the top views, droplets are elongated only in the flow direction (z-direction), because these droplets are near the center in the width direction (x-direction).

### 3.2. Top Focusing Position Splitting and Shifting

**Figure 3:**
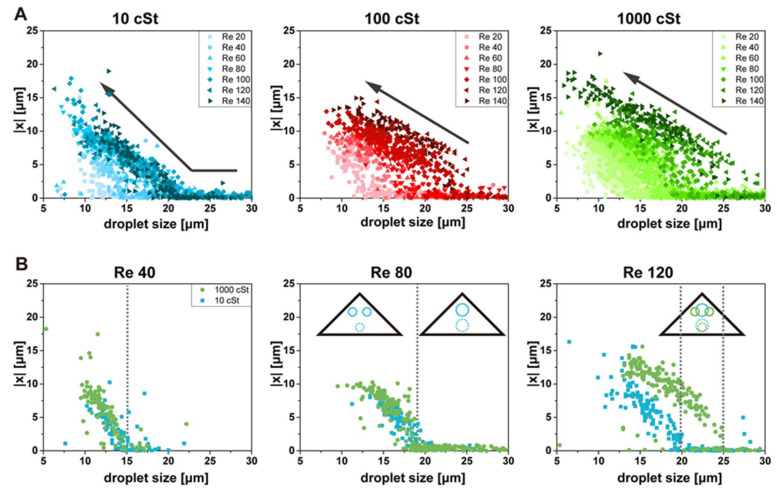
Changes of the top-view focusing positions depending on size, Re, and deformability. (**A**) The size of the droplet is plotted against the x-position of the focusing position. Smaller droplets focus further away from the channel centerline with given Re and deformability. (**B**) Comparison of 10 cSt and 1000 cSt droplets at three different Re. The threshold of the focusing position splitting is indicated with gray dotted lines.

First, we have analyzed the changes of the top focusing position in the top view with varying Re and deformability. The size and x-position of droplets are measured from the top-view images, and the x-position of the droplet is plotted against its size, as shown in Figure 3. Droplets in the top-view images can be either at the top focusing positions or at the bottom focusing position, which should be distinguished to collect and analyze the data for the top focusing positions only. We used an objective lens (S Plan Fluor ELWD 20x, Nikon, Tokyo, Japan) with the depth of the focus of approximately 7 μm. The images of out-of-focused droplets have a large blur at the boundary, which can be easily distinguished from the images of particles near the focal plane. We adjust the focal plane away from the channel bottom to eliminate the droplets focused at the bottom focusing positions. From now on, the term ‘focusing positions’ will be used for the top focusing positions unless the bottom focusing position is mentioned specifically. The bottom focusing position is occupied under all conditions studied here. The bottom focusing position does not show noteworthy changes with varying Re or deformability.

Figure 3A shows that the trend of the focusing position change of droplets, which generally agrees with the trend from solid particles. The x-position of the focusing position and the size of the droplet have strong negative correlations (arrows), which also depends on Re. At a given Re, droplets larger than a specific threshold size are focused at the channel center (x = 0). Splitting and shifting of the focusing position can be observed with the decrease of droplet size. The threshold size of the splitting depends on the Re and viscosity of droplets. The data points are further away from the origin for larger Re, and the threshold size of splitting becomes larger with increasing Re. The same trend has been found for the inertial focusing of solid particles [13].

Interesting results are observed when the deformability of droplets is varied. The focusing position changes with the variation of Re show a noticeable difference for the droplets with different viscosity. The data points for the lower viscosity droplets are less scattered than those of the higher viscosity droplets. In other words, the change of the focusing position with Re is smaller for more deformable particles than less deformable particles. The increase of Re beyond 100 does not make much difference for 10 cSt droplets; the top focusing position of droplets does not split with the further increase of Re when the size is larger than approximately 25 μm. On the contrary, for 100 and 1000 cSt droplets, the threshold size of splitting keeps increasing with the increase of Re.

In Figure 3B, we compare the focusing positions of droplet 10 cSt and 1000 cSt at three different Re. For the comparison of Re = 20 and 80, the overall data points shift to the right with the increase of Re. No significant difference in focusing position is found for droplets with different viscosities (10 cSt, 1000 cSt) at these Re. The threshold of splitting is increased from approximately 15 μm to 19 μm for both types of droplets. With further increase of Re up to 120, the data points for 10 cSt barely change, and the threshold size of splitting remains similar to the case of Re = 80. On the other hand, the data points still shift for 1000 cSt, and the threshold size of splitting changes further to approximately 25 μm.

**Figure 4:**
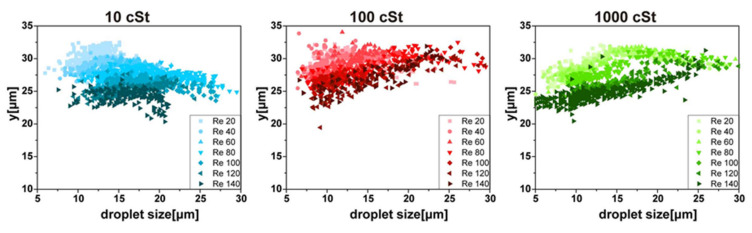
Changes of the side-view focusing positions depending on size, Re, and deformability. Generally, the smaller droplets focus further away from the vertex.

Similar to observation from the top view, the size and the y-position of the droplets are measured in the side view with varying Re and deformability, as shown in Figure 4. The focusing position variation depending on the droplet size is smaller in the side view (height direction) than in the top view (width direction). The y-position of the focusing position is mostly between 20 and 35 μm, while the x-position varies from 0 to 20. This difference suggests that the shifting of the focusing position is not parallel along the sidewalls. It will become apparent in the next section with focusing position plots in the cross-section. Generally, the smaller droplets are further away from the vertex than the larger droplets, which agrees with top-view observation. This size dependence is more substantial for droplets of smaller deformability at large Re. Other than these general findings, the changes in the side view show more complexity with varying Re and deformability. For 10 cSt droplets, the focusing position barely changes with droplet size. On the other hand, the changes with Re are evident. The focusing position shifts to smaller y-position with varying Re, which is common for all deformability. For less deformable droplets, the size-dependent focusing position change becomes more evident with increasing Re; the focusing position for smaller droplets is further away from the vertex than that of larger droplets.

**Figure 5:**
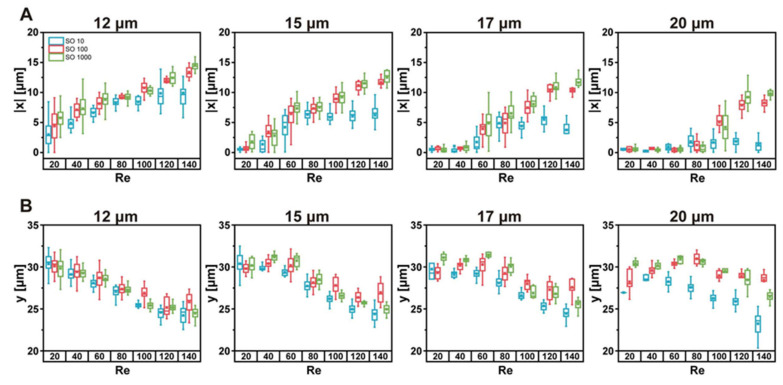
Comparison of focusing position changes depending on deformability and Re at fixed droplet size. (**A**) Top view and (**B**) side view.

Figure 5 shows the comparison of the focusing positions for various Re and deformability. The x-position data and y-position data are collected for droplets of 4 different sizes 12, 15, 17, and 20 μm with ±1 μm range Figure 5. In the top view, the top focusing position splits and shifts, moving away from the centerline, as Re increases, as shown in Figure 5A. Splitting starts at larger Re for larger particles. For 12 μm droplets, no focusing at the center is observed in the whole Re range. This trend is similar to previous studies with solid particles [12,13]. It appears that the focusing positions of 100 cSt and 1000 cSt droplets are similar to each other, but the focusing position of the 10 cSt droplets shifts less than the others, especially at high Re. Such similarity between 100 cSt droplets and 1000 cSt droplets may result from a similar degree of deformation, as shown in Figure 2. Different shifting trends between 10 cSt droplets and others can also be found from the side view (Figure 5B). The difference is small for smaller droplet size and becomes more significant with the increase of the droplet size. For 20 μm droplets, in particular, the focusing position of 10 cSt droplets can be clearly distinguished from the others when Re >60. Interestingly, the reversing of the shifting direction is found for large droplets. For 10 and 100 cSt droplets with 20 μm size, the focusing position appears to be shifting toward the vertex (away from the channel center) with Re = 20–60. Then, the shifting direction changes with the further increase of Re. A similar trend can also be found from less deformable and smaller size droplets.

### 3.3. Focusing Position Changes in Cross-Section

**Figure 6:**
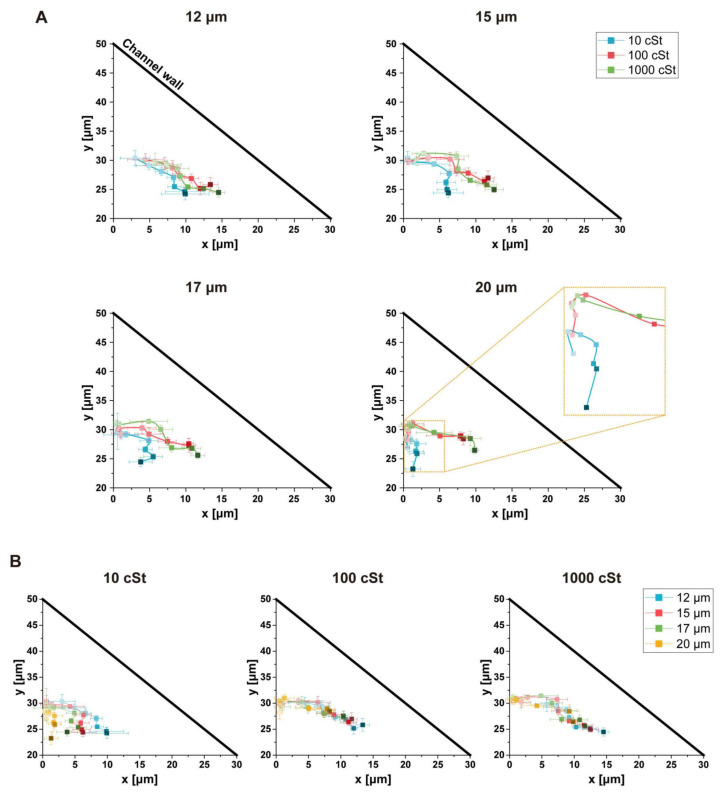
Focusing position changes in cross-section with increasing Re from 20 to 140. (**A**) Comparison of the paths of the focusing position shift for droplets with different deformability. The paths for 10 cSt droplets are distinguishable from the other droplets, and the difference becomes bigger for larger droplet sizes. (**B**) Comparison of the paths of the focusing position shift for droplets with different sizes. The paths are closer to the channel center for larger droplet size in the case of 10 cSt droplets. The paths mostly overlap in a single curve in the case of 100 and 1000 cSt droplets.

In Figure 6, the change of the focusing position from the top view and side view are combined and plotted in channel cross-sections as a function of Re. These graphs show the paths of focusing position shift with varying Re in the channel cross-section. First, we plotted the focusing positions in the cross-section for each droplet size to compare the effect for deformability, as shown in Figure 6A. Droplets of different deformability are shown with different color and points; the darker color represents a higher Re. A common trend can be found for all sizes; focusing positions locate near the vertex at low Re and shift away from the vertex with increasing Re. In the meanwhile, the shifting direction is strongly dependent on the deformability and droplet size. For smaller droplets, the trends of the shifting do not vary significantly depending on the droplet deformability. However, the difference becomes evident with increasing size. For example, the shifting directions of 10 cSt droplets and 1000 cSt droplets are entirely different for 20 µm droplets; the focusing position of 10 cSt droplets shifts vertically down toward the channel center, while the focusing position of 1000 cSt droplets shifts along the sidewall. Generally, 100 cSt droplets and 1000 cSt droplets show a similar trend, and the shifting is along the sidewall for all size droplets. As mentioned in the previous section, the direction of the focusing position shift is in the + y-direction in the case of small Re and large droplet size. The magnified view for the 20 µm droplets shows this change of shifting direction.

Figure 6B shows the comparison between different size droplets with fixed viscosity. For the 100 cSt and 1000 cSt droplets, the paths of the focusing position shift for different sizes mostly overlap in a single curve along the sidewall. The focusing positions start from the middle of the channel (x = 0) and shift in the positive x-direction in the beginning. Then, the focusing positions shift nearly parallel to the sidewall. The shifting direction is in the positive y-direction toward the vertex for relatively larger size droplets at low Re. Otherwise, the splitting and shifting of the focusing position are similar to that which is found for solid particles. For the 10 cSt droplets, on the contrary, the paths of the focusing position shift show an explicit dependency on droplet size. The paths are closer to the channel center for larger droplets, while the paths for smaller droplets are near the sidewall. The deformability-induced lift force acts in a direction away from the channel walls. The paths of the inertial focusing position shifts are expected to be closer to the channel center for more deformable droplets, which become more significant with larger droplets. As seen in Figure 2, larger droplets of 10 cSt viscosity deform more significantly, which supports the observation that the large focusing position changes closer to the channel center.

### 3.4. Comparison with Solid Particles and Feasibility of Deformability-Based Separation

**Figure 7:**
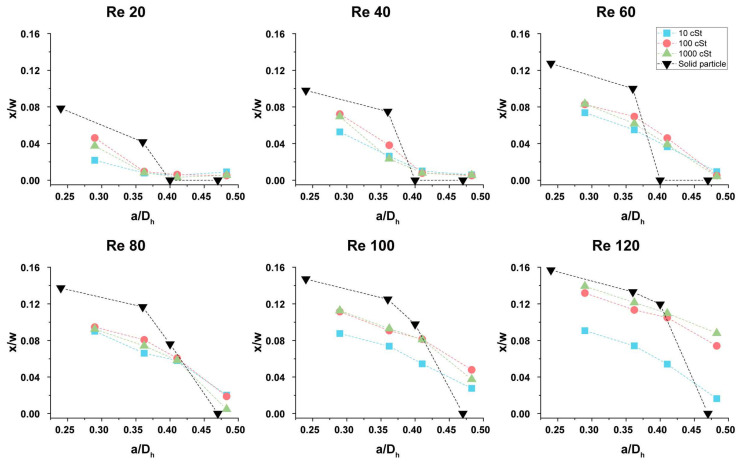
Comparison of the focusing positions of droplets and solid particles. Solid particles are generally further away from the channel centerline than the droplets.

The inertial focusing of droplets cannot precisely represent the inertial focusing of cells, because the nature of the deformation of cells and their interaction with fluids is more complex than that of viscous droplets. However, the analysis of the effect of deformability-induced lift force by comparison of solid particles and deformable droplets would provide insight over the difference of separation efficiency between solid particles and cells. Figure 7 shows the focusing positions of the droplets from Figure 5A and the focusing positions of solid particles from the previous study [13]. The dimensionless position (*x/w*, where *w* is the channel width) and dimensionless size (*a/D_h_*) are used to compare the results. Typical inertial separation devices use an expanding channel near the outlets, and the x-position of the focusing position mostly determines the separation efficiency. Therefore, we compare the x-position of the focusing positions. Figure 7 shows that the focusing position shift of solid particles is generally larger than that of deformable droplets. For small size, solid particles focus further away from the channel center than droplets. Among droplets, the highest viscosity droplets focus furthest away from the channel center. Interestingly, the onset of splitting is smaller for solid particles than droplets with relatively large Re; the large *a/D_h_* = 0.47 solid particles are focused at the channel center even at Re = 120, while the large *a/D_h_* = 0.5 droplets are focused on the sidewall when Re > 100. Further in-depth studies are needed to understand this unexpected transition. Other than this exception, the deformability of particles works as a suppressor for the splitting and shifting of the focusing position.

**Figure 8:**
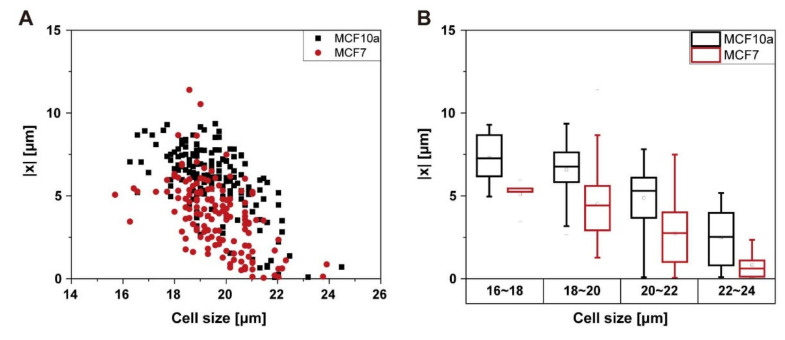
Focusing positions of cell lines with different deformability (**A**) More deformable MFC7 cells focus closer to the channel center. (**B**) Comparison of focusing positions after sorting with cell size.

Deformability is a useful cell biomarker that can be used for the separation of cell types or phenotypes. For example, cancer cells are generally more deformable than healthy cells [47,48], and the cell separation based on deformability has been demonstrated with an inertial microfluidic device with a rectangular cross-section channel [18] and spiral channel [39]. Hur at al. showed that the deformability-induced lift force significantly changed the focusing positions depending on cell types, which allows the enrichment of specific cell types. We expect that it is also possible to use the inertial focusing in a triangular channel for deformability-based cell separations. Here, we measure the focusing positions of the MCF7 and MCF10a (Figure 8). MCF7 is a breast cancer cell line, and MCF 10A is a non-tumorigenic epithelial breast cell line. It is known that MCF7 cells have larger deformability than MCF10A [22,49]. The focusing positions of MCF7 and MCF10a cells are plotted against their size at Re 140 (Figure 8A). The focusing positions of MCF 7 are generally closer to the channel center than MCF10a if the size is the same. It can be more clearly seen in Figure 8B, where the focusing positions of cells with similar size are compared. This observation agrees with the result from droplets; the top focusing position of more deformable droplets splits with a smaller threshold size, and the focusing positions are closer to the channel center. Although there is a rather large overlap of the focusing positions for the two cell types, the results suggest that it is feasible to achieve the separation or enrichment of the cells based on their deformability.

The inertial focusing and separation of cells using a triangular channel is difficult to predict because it involves a complex function with multiple variables, including size, Re, and deformability. For instance, red blood cells and white blood cells are smaller than typical cancer cells, but they are generally more deformable. The splitting of the focusing position occurs at lower Re for smaller particles, while higher deformability suppresses the splitting of the focusing position, which would lead to a reduced cell-separation efficiency. In fact, this observation may explain the lower separation efficiency in the case of cell separations than particle separations using a triangular channel inertial microfluidic device [13].

## 4. Conclusions

The inertial focusing position of droplets in a triangular channel is observed at the top and side view with varying Re, droplet deformability, and droplet size. The splitting and shifting of the top focusing position in triangular channels generally depend on the particle size and Re. This study reveals that deformability also affects this focusing position changes. The general dependency of the focusing position splitting and shifting on Re and droplet size is similar to that of solid particles; the focusing position splits and shifts with increasing Re and decreasing particle size. For the droplets of larger deformability, the threshold size of the focusing position splitting becomes larger, which leads to smaller focusing position shifts for more deformable droplets with the same size. The focusing positions of the 10 cSt droplets are clearly distinguishable, while the focusing positions of the 100 and 1000 cSt droplets are similar. Notably, the path of the focusing position shift in the cross-section is vertically toward the channel center with 10 cSt droplets with large size, indicating that large deformability-induced lift force dominates over the other lift forces that lead to shifting along the sidewalls. Although the physics of the inertial focusing of droplets have a fundamental difference with the inertial focusing of cells, the investigation of the deformability-induced lift force in a triangular channel can allow us to better understand and engineer the focusing and separation of the cells in triangular channel systems. As Re increases, the focusing positions of droplets with different deformability become distinguishable. As shown with MCF7 and MCF10a cells, triangular channels are expected to allow deformability-based inertial cell separation.
